# Urate and Its Transgenic Depletion Modulate Neuronal Vulnerability in a Cellular Model of Parkinson's Disease

**DOI:** 10.1371/journal.pone.0037331

**Published:** 2012-05-14

**Authors:** Sara Cipriani, Cody A. Desjardins, Thomas C. Burdett, Yuehang Xu, Kui Xu, Michael A. Schwarzschild

**Affiliations:** Neurology Department, MassGeneral Institute for Neurodegenerative Disease, Massachusetts General Hospital, Boston, Massachusetts, United States of America; Virginia Commonwealth University, United States of America

## Abstract

Urate is a major antioxidant as well as the enzymatic end product of purine metabolism in humans. Higher levels correlate with a reduced risk of developing Parkinson's disease (PD) and with a slower rate of PD progression. In this study we investigated the effects of modulating intracellular urate concentration on 1-methyl-4-phenyl-pyridinium (MPP^+^)-induced degeneration of dopaminergic neurons in cultures of mouse ventral mesencephalon prepared to contain low (neuron-enriched cultures) or high (neuron-glial cultures) percentage of astrocytes. Urate, added to the cultures 24 hours before and during treatment with MPP^+^, attenuated the loss of dopaminergic neurons in neuron-enriched cultures and fully prevented their loss and atrophy in neuron-astrocyte cultures. *Exogenous* urate was found to increase intracellular urate content in cortical neuronal cultures. To assess the effect of reducing cellular urate content on MPP^+^-induced toxicity, mesencephalic neurons were prepared from mice over-expressing urate oxidase (UOx). Transgenic *UOx* expression decreased *endogenous* urate content both in neurons and astrocytes. Dopaminergic neurons expressing UOx were more susceptible to MPP^+^ in mesencephalic neuron-enriched cultures and to a greater extent in mesencephalic neuron-astrocyte cultures. Our findings correlate intracellular urate content in dopaminergic neurons with their toxin resistance in a cellular model of PD and suggest a facilitative role for astrocytes in the neuroprotective effect of urate.

## Introduction

Urate (2,6,8-trioxy-purine; a.k.a. uric acid) is generated within cells from the breakdown of purines. In most mammals urate is converted to allantoin by uricase (urate oxidase; UOx) [1], an enzyme primarily expressed in the liver [2]. In humans and apes, uricase is not synthesized due to the sequential non-sense mutations of its gene (*UOx*) that occurred during hominoid evolution [3]–[5]. Thus, in humans urate is the end product of the purine catabolism and achieves concentrations approaching the limit of solubility, which are more than fifty times higher than those in other mammals [6]. Due its high levels and radical scavenging properties [7]–[9] urate is considered a major antioxidant circulating in humans. It may have played a facilitative role in human evolution as was initially proposed based on putative central nervous system benefits [10]–[12] and later based on its antioxidant properties – perhaps to have partially compensated for the lost of the capability of synthesizing ascorbate [7], [13]. Urate's antioxidant proprieties have been extensively characterized *in vitro* where it was found to be a peroxynitrite scavenger [14] and to form stable complex with iron ions, reducing their oxidant potential [15].

Identification of these antioxidant proprieties of urate, together with evidence that oxidative damage plays a critical role in the neurodegeneration of PD, raises the possibility that urate may protect from the development of the disease. Prompted further by post-mortem evidence that the urate levels in midbrain and striatum of PD patients are reduced compared to those of control brains [16], epidemiological and clinical cohorts were investigated for a possible link between urate level and the risk of PD or the rate of its progression. Several studies found lower blood urate concentration in healthy individuals to be a reproducible risk factor for developing PD later in life [17]–[19]. Furthermore, among those already diagnosed with PD, lower serum levels were consistently associated with a more rapid clinical and radiographic progression of PD [20]–[22], suggesting urate may be a prognostic biomarker in PD. In addition, an inverse correlation between serum urate level and disease duration has been reported in PD and raises the possibility that urate may also be a marker of disease stage [23], though falling urate may simply reflect the weight loss that accompanies disease duration.

A causal basis for the link between urate and favorable outcomes in PD is supported by the neuroprotective properties of urate in models of PD. Presumably by reducing ROS levels, urate can prevent cellular damage and increase cell viability in *in vitro* models of toxicant-induced or spontaneous cell death [24]–[27]. Moreover, urate increased cell survival in MPP^+^-treated cell cultures [28] and prevented dopaminergic neuron loss in a rodent model of PD [29].

MPP^+^ (1-methyl-4-phenylpyridinium) is the toxic metabolite of MPTP (1-methyl-4-phenyl-1,2,3,6-tetrahydropyridine) [30], an agent shown to induce a parkinsonian condition in humans [31]. MPP^+^ is generated in astrocytes and up-taken by dopamine transporter into dopaminergic neurons [32]. Within the cells, MPP^+^ can induce the irreversible inhibition of complex I activity, failure of ATP synthesis and cell death [33], [34]. In this study we assessed whether modulating urate level in primary dopaminergic neurons affects their vulnerability to MPP^+^ toxicity in the presence of a low or high percentage of astrocytes.

## Results

### Urate prevents dopaminergic neuron loss in MPP^+^-treated cultures

**Figure 1:**
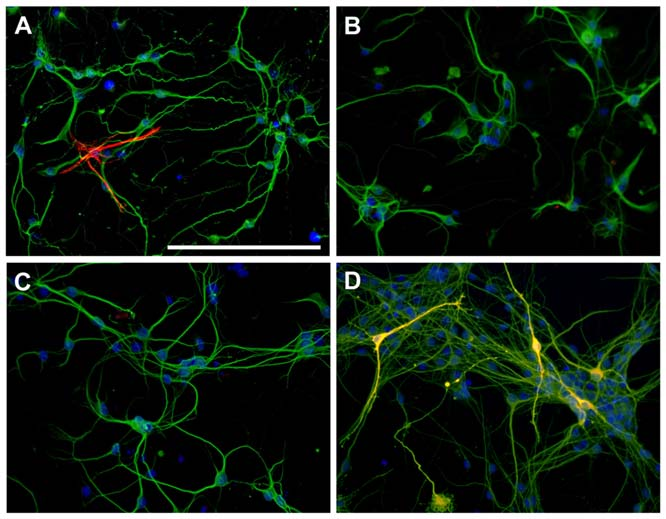
Cellular composition of neuron-enriched cultures. Composite fluorescence photomicrographs of neuron-enriched cultures that were immuno-stained with *A*–*D*) the neuronal marker MAP-2 (green) together with *A*) astrocyte marker GFAP (red) or *B*) the microglia marker CD11b (red, not detected) or *C*) the oligondendrocyte marker CNPase (red, not detected) or *D*) the dopaminergic neuron marker TH (yellow). Nuclei were counterstained with DAPI; scale bar length represents 100 µm.

**Figure 2:**
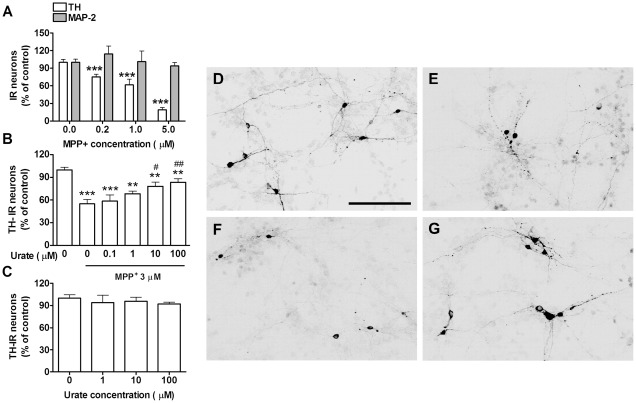
Urate's protective effect on dopaminergic neurons in neuron-enriched cultures. *A*) MPP^+^ concentration-dependent effect on dopaminergic and total neuron viability expressed respectively as percentage of TH- and MAP-2-IR cell number in comparison to control cultures (n = 5). *B*) Urate concentration-dependent effect on TH-IR cell number in 3 µM MPP^+^-treated cultures (n = 7). *C*) Lack of urate effect at any concentration on TH-IR neuron number in control (MPP^+^-untreated) cultures (n = 5). Photomicrographs show TH-IR neurons in *D*) control cultures, *E*) MPP^+^/0 urate-treated cultures, *F*) MPP^+^/0.1 urate-treated cultures and *G*) MPP^+^/100 µM urate-treated cultures. Scale bar = 50 µm. One-way ANOVA followed by Newman-Keuls test: **P<0.01, ***P<0.001 *vs* 0 MPP^+^ value; ^#^ *P*<0.05, ^##^ *P*<0.01 *vs* MPP^+^/0 urate value.

To identify an MPP^+^ concentration with selective toxicity for dopaminergic neurons, mesencephalic neuron-enriched cultures (Fig. 1*A*–*D*) were treated for 24 hours with increasing concentrations of MPP^+^. Toxicant treatment reduced the number of dopaminergic neurons, which were identified by their immunoreactivity for tyrosine hydroxylase (TH), in a concentration-dependent manner (*P*<0.0001). There was no change in the total number of neurons, which were scored as microtubule-associated protein 2-immunoreactive (MAP-2-IR) cells (Fig. 2*A*), due to the selectively toxic effect of MPP^+^ on dopaminergic neurons and their low number in ventral mesencephalon cultures (2–3% of MAP-2-IR cells; see also Materials and Methods). To assess the effect of urate on dopaminergic neuron viability, neuron-enriched cultures were pretreated with urate 24 hours before and during exposure to 3 µM MPP^+^. In MPP^+^-treated cultures urate increased TH-IR viability over a concentration range of 0.1–100 µM (*P*<0.0001). The maximum effect was achieved at 100 µM with a 51% increase in TH-IR cell number in comparison to cells treated with MPP^+^ only (*P*<0.01). Half-maximally effective concentration (EC_50_) was achieved at a concentration of 1 µM [95*%* confidence interval (95%CI): 0.096–5.9] (Fig. 2B, D–*G*). Urate on its own produced no significant effect on dopaminergic neuron viability (Fig. 2*C*).

**Figure 3:**
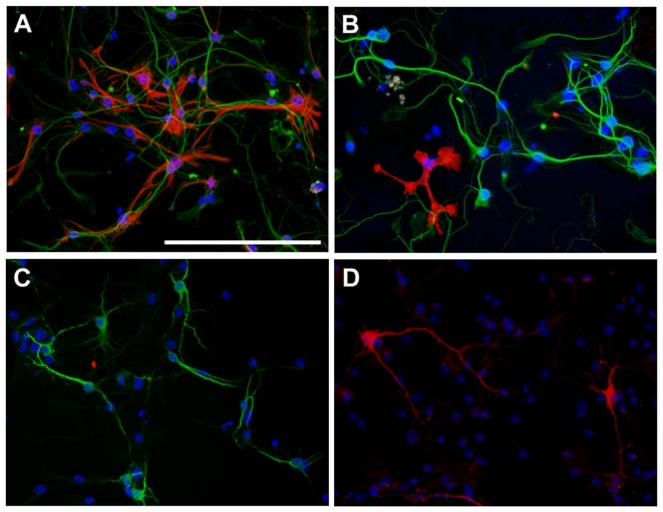
Cellular composition of neuron-astrocyte cultures. Composite fluorescence photomicrographs of neuron-astrocyte cultures that were immuno-stained with *A*–*C*) the neuronal marker MAP-2 (green) together with *A*) astrocyte marker GFAP (red) or *B*) the microglia marker CD11b (red) or *C*) the oligondendrocyte marker CNPase (red, not detected). *D*) Dopaminergic neurons were stained with the dopaminergic neuron marker TH (red). Nuclei were counterstained with DAPI; scale bar is 100 µm.

**Figure 4:**
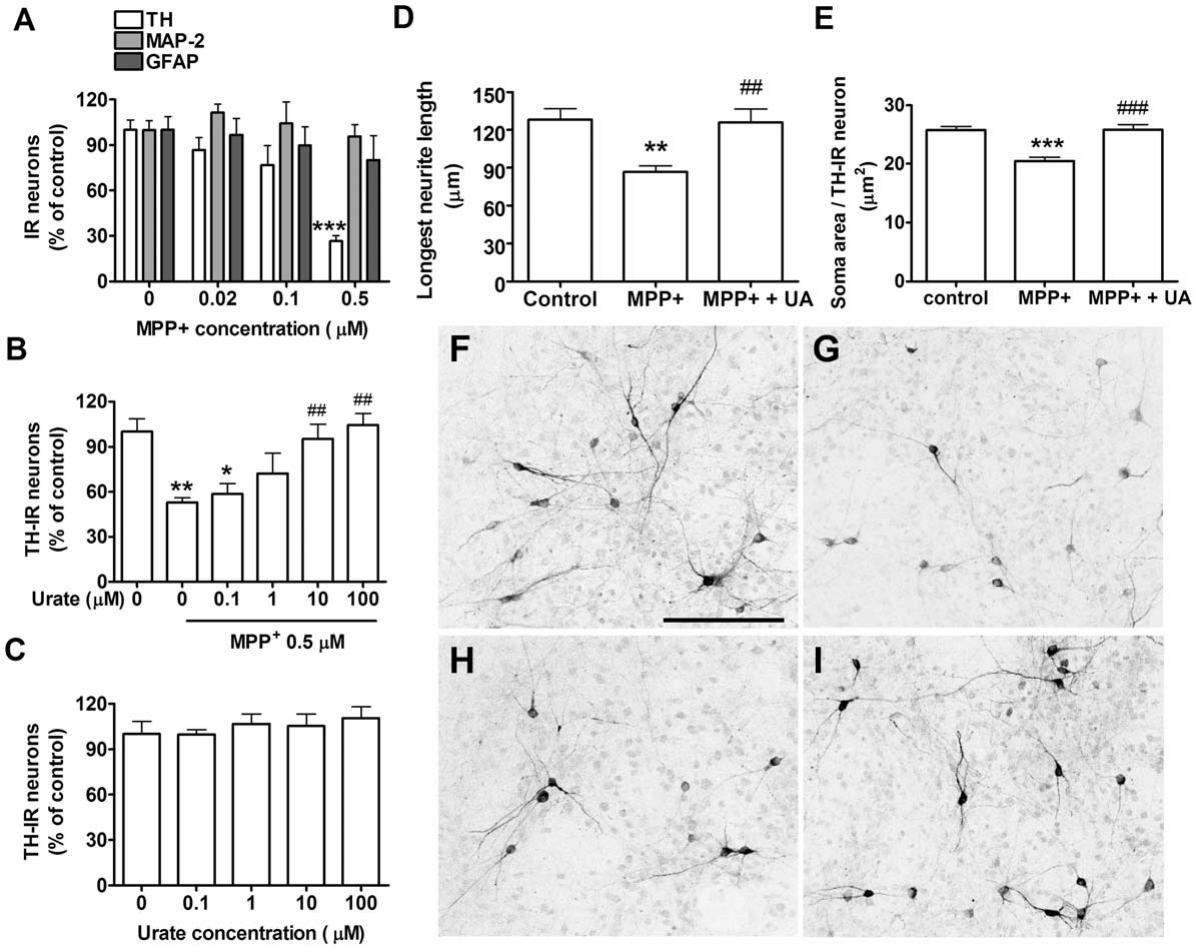
Urate's protective effect on dopaminergic neurons in mixed cultures. *A*) MPP^+^ concentration-dependent effect on dopaminergic neuron, total neuron and astrocyte viability, expressed as percentage of TH-IR, MAP-2-IR and GFAP-IR cell number, respectively, in comparison to control cultures (n = 4). *B*) Urate concentration-dependent effect on TH-IR cell number in 0.5 µM MPP^+^-treated cultures (n = 5). *C*) Lack of effect of urate at any concentration on TH-IR cell number (n = 5). Urate (100 µM) effects on reductions in *D*) longest neurite length and *E*) soma size in MPP^+^ urate-treated TH-IR neurons. Photomicrographs show TH-IR neurons in *F*) control cultures, *G*) MPP^+^/0 urate-treated cultures and *H*) MPP^+^/0.1 urate-treated cultures and *I*) MPP^+^/100 µM urate-treated cultures. Scale bar = 50 µm. One-way ANOVA followed by Newman-Keuls test: *P<0.05, **p<0.01, ***P<0.001 *vs* 0 MPP^+^ value, ^##^ *P*<0.01 and ^###^ *P*<0.001 *vs* MPP^+^/0 urate value.

Previous data [35] have shown that urate's protective effect against toxin-induced neuronal cell death can be dependent on the presence of astrocytes in cultures. In our study urate treatment in neuron-enriched cultures only partially attenuated MPP^+^ toxicity on dopaminergic neurons. To assess whether astrocytes might potentiate the protective effect of urate in our cells, urate was tested in MPP^+^-treated mixed neuron-astrocyte cultures (Fig. 3*A*–*D*). To obtain selective degeneration of dopaminergic neurons without toxic effect on non-TH-IR cells, cultures were treated with relatively low concentrations of MPP^+^ for four days as previously described [36]. MPP^+^ induced selective loss of TH-IR neurons in a concentration-dependent manner (*P* = 0.0005) with no statistically significant effect on MAP-2-IR or glial fibrillary acid protein-immunoreactive (GFAP-IR) cells (Fig. 4*A*). To assess the effect of urate, neuron-astrocyte cultures were pretreated with urate 24 hours before and during exposure to 0.5 µM MPP^+^. Urate increased the number of TH-IR neurons over a concentration range of 0.1–100 µM (*P*<0.0001). The maximum effect was seen at 100 µM with a 97% increase in the number of TH-IR neurons in comparison to cultures treated with MPP^+^ only (*P*<0.01; Fig. 4*B, F–I*), corresponding to a complete blockade of MPP^+^ toxicity. Urate on its own did not affect TH-IR cell number (Fig. 4*C*). No statistically significant difference was seen at the estimated EC_50_'s for urate in neuron-enriched and neuron-astrocytes cultures (∼1 µM in both; F_1,53_ = 0.01, *P* = 0.9).

### Urate prevents MPP^+^-induced atrophic changes in dopaminergic neurons

To assess whether the protective effect of urate on neuronal viability correlates with an improvement in toxin-induced cellular atrophy, neurite length and soma size were analyzed in neuron-astrocyte cultures. In MPP^+^-treated cultures TH-IR cells showed shorter neurites (−32%, *P*<0.01) and smaller soma area (−20%, *P*<0.001) in comparison to control cells (Fig. 4*D and E*, respectively). The concentration that fully protected against dopaminergic neuron loss, 100 µM urate, prevented the decrease in neurite length (*P*<0.01) and soma size (*P*<0.001) in TH-IR neurons (Fig. 4*D*–*E*).

### *Exogenous* urate raises its intracellular level

**Figure 5:**
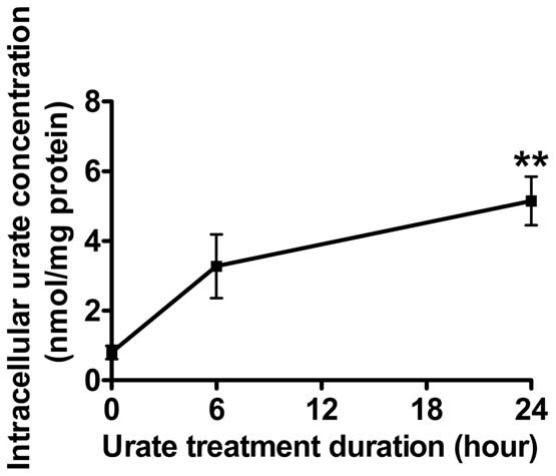
Urate accumulation in cortical neurons. *A*) Time-dependent effect of 100 µM *exogenous* urate on its intracellular content in primary cortical neurons. One-way ANOVA followed by Newman-Keuls test: **P<0.01.

To assess whether urate's protective effects are associated with an increase in its intracellular content, neuron-enriched cultures were treated with *exogenous* urate for 0, 6 and 24 hours. In order to obtain the large number of neurons required for intracellular analyte measurements, cultures were prepared from the mouse cortex for this assay. Urate content in neurons increased in a time-dependent manner with about 4 fold increase at 24 hours of treatment (*P* = 0.002) (Fig. 5*A*), the time at which MPP^+^ would be added to the cultures. *Exogenous* urate did not affect the concentration of any measured urate precursor (adenosine, inosine, hypoxanthine and xanthine) within neurons (unpublished data). Similar results were obtained in astrocyte-enriched cultures (unpublished data).

### Transgenic *UOx* expression lowers intracellular urate

**Figure 6:**
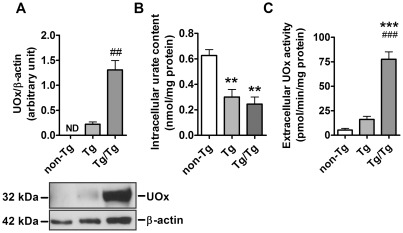
Characterization of non-Tg, Tg and Tg/Tg cortical neuron-enriched cultures. *A*) Western blot and graph showing UOx expression in wild-type (non-Tg) and UOx-expressing neurons (Tg and Tg/Tg) normalized to the β-actin level. Note that UOx was not detected in wild-type neurons (n = 3). *B*) Effect of UOx expression on intracellular urate content in neurons normalized to the protein level (n = 3). C) UOx activity in the media of non-Tg and UOx-expressing neurons (n = 6). Student's *t* test: ^##^ *P* = 0.005 *vs* Tg value; one-way ANOVA followed by Newman-Keuls test: **P<0.01, ***P<0.001 *vs* non-Tg value and ^###^ *P*<0.001 *vs* Tg value.

**Figure 7:**
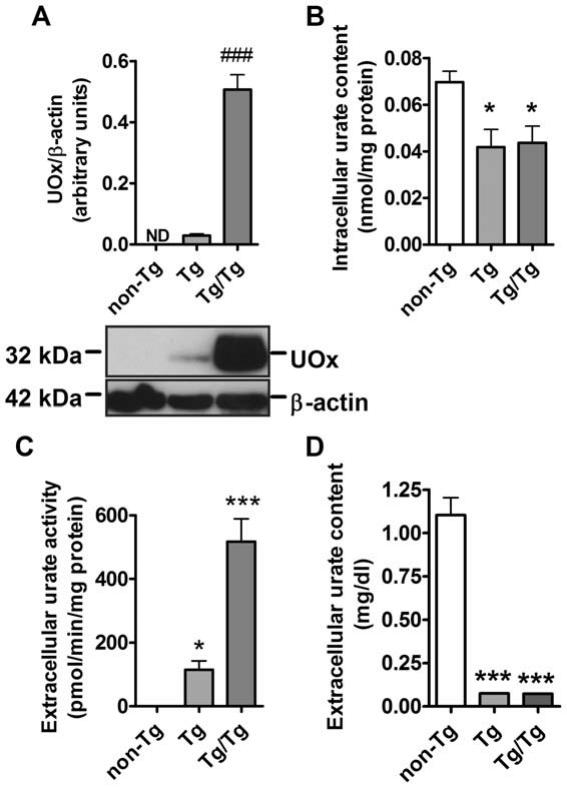
Characterization of non-Tg, Tg and Tg/Tg cortical astrocyte-enriched cultures. *A*) Western blot and graph showing UOx immunostaining in non-Tg and UOx-expressing astrocytes (Tg and Tg/Tg) normalized to the β-actin level. Note that UOx was not detected in non-Tg astrocytes (n = 7). *B*) Effect of UOx expression on the intracellular urate content normalized to the protein level (n = 5). *C*) UOx activity in the media of non-Tg and UOx-expressing astrocytes (n = 9). *D*) Effect of UOx expression on extracellular urate concentration in astroglial cultures (n = 6). Some error bars are not visible because of their small size. Student's *t* test: ^###^ *P*<0.0001 *vs* Tg value. One-way ANOVA followed by Newman-Keuls test: *P<0.05, ***P<0.001 *vs* non-Tg value.

To assess whether intracellular urate content affects dopaminergic neuron resistance to MPP^+^ we prepared ventral mesencephalon cultures from a mouse line expressing transgenic uricase (*UOx*) [37], the enzyme that converts urate to allantoin. Intracellular urate content was measured in cortical neurons and astrocytes prepared from non-transgenic *UOx* (non-Tg), hemizygous transgenic *UOx* (Tg) and homozygous (double) transgenic *UOx* (Tg/Tg) mice. In Tg/Tg neurons UOx expression was about 6 times higher than in Tg neurons as assessed by western blotting; in non-Tg neurons UOx was not detected (Fig. 6*A*). UOx expression reduced intracellular urate content by 50% (*P*<0.01) and 60% (*P*<0.01) in Tg and Tg/Tg neurons, respectively (Fig. 6*B*). UOx activity was significantly increased in Tg/Tg (p<0.001) but not in Tg cell medium in comparison to non-Tg samples (Fig. 6*C*). In Tg/Tg astrocytes intracellular UOx expression was about 15 times higher than in Tg astrocytes; in non-Tg astrocytes UOx was not detected (Fig. 7*A*). UOx expression reduced intracellular urate content by 30% both in Tg and Tg/Tg (*P*<0.01) astrocytes (Fig. 7*B*). UOx activity was detected in the cell media of Tg and Tg/Tg astrocytes (Fig. 7*C*) where medium urate concentration was significantly reduced in comparison to that from non-Tg astrocytes (*P*<0.001) (Fig. 7*D*).

### Transgenic UOx reduces neuronal resistance to MPP^+^ toxicity

**Figure 8:**
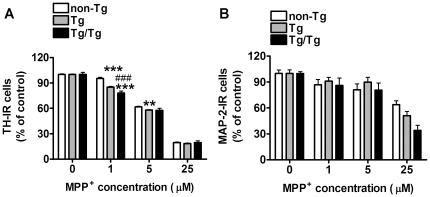
MPP^+^ effect on non-Tg, Tg and Tg/Tg neuron-enriched cultures. *A*) MPP^+^ effect on TH-IR cell number in non-Tg (n = 18), Tg (n = 35) and Tg/Tg (n = 8) neuronal cultures. *B*) MPP^+^ effect on MAP-2-IR cell number in non-Tg (n = 18), Tg (n = 35) and Tg/Tg (n = 8) cultures. *C*) Two-way ANOVA followed by Bonferroni multiple comparison test: **P<0.01, ***P<0.001 *vs* respective non-Tg value; ^###^ *P*<0.01 vs respective Tg value.

To determine whether the enzymatic reduction of intracellular urate exacerbates dopaminergic susceptibility to MPP^+^, neuron-enriched ventral mesencephalon cultures from non-Tg, Tg and Tg/Tg mice were treated with increasing concentrations of toxin for 24 hours. Two-way ANOVA showed that both genotype (F_2,232_ = 24.61, *P*<0.0001) and MPP^+^ concentration (F_2,232_ = 312.64, *P*<0.0001) affected the number of TH-IR neurons, and found significant interaction between these two factors (F_2,232_ = 13.82, *P*<0.0001). Dopaminergic viability was reduced in UOx expressing cultures in comparison to non-Tg cultures with a maximum effect at 1 µM MPP^+^, which further reduced TH-IR neuron number by 10% and 18% in Tg and Tg/Tg cultures compared to non-Tg cultures, respectively (Fig. 8*A*). The EC_50_ for MPP^+^ was 5.2 µM (95%CI: 2.8–9.7 µM) in non-Tg cultures, 3.9 µM (95%CI: 2.4–6.4 µM) in Tg and 2.5 µM (95%CI: 0.9–6.8 µM) in Tg/Tg without statistically significant difference among genotypes (F_2,232_ = 0.5612, *P* = 0.57).

Two-way ANOVA of MPP^+^-toxicity on MAP-2-IR cell number revealed significant effect of MPP^+^ concentration (F_2,199_ = 28.47, *P*<0.0001), but neither a significant effect of genotype (F_2,199_ = 1.64, *P* = 0.20) nor a significant interaction between these two factors (F_2,199_ = 1.20, *P* = 0.31) (Fig. 8*B*).

**Figure 9:**
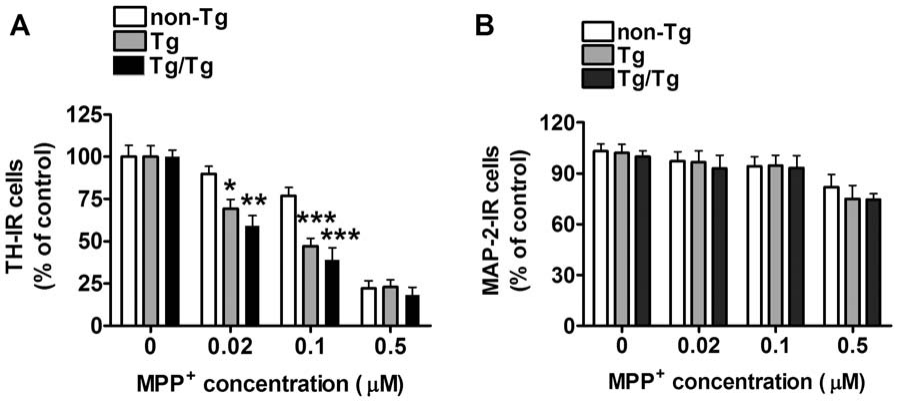
MPP^+^ effect on non-Tg, Tg and Tg/Tg mixed neuron-astrocyte cultures. *A*) MPP^+^ effect on TH-IR cell number in non-Tg (n = 18), Tg (n = 34) and Tg/Tg (n = 22) neuronal cultures. *B*) MPP^+^ effect on MAP-2-IR cell number in non-Tg (n = 18), Tg (n = 34) and Tg/Tg (n = 22). Two-way ANOVA followed by Bonferroni multiple comparison test: *P<0.05, **P<0.01, ***P<0.001 *vs* respective non-Tg value.

To assess whether reducing basal urate levels in both, neurons and astrocytes, exacerbated the UOx effect on MPP^+^-induced toxicity, we treated neuron-astrocyte cultures with MPP^+^ for four days as mentioned above. Two-way ANOVA revealed significant effects of both genotype (F_2,284_ = 10.09, *P*<0.0001) and MPP^+^ concentration (F_2,284_ = 96.36, *P*<0.0001) on the number of TH-IR neurons and a significant interaction between genotype and MPP^+^ concentration (F_2,284_ = 3.01, *P* = 0.007) (Fig. 9*A*). Dopaminergic viability was reduced in UOx expressing cultures in comparison to non-Tg cultures with a maximum effect at 0.1 µM MPP^+^, which further reduced TH-IR neuron number by 39% and 49% in Tg and Tg/Tg cultures compared to non-Tg cultures, respectively. The EC_50_ for MPP^+^ was 0.11 µM (95%CI: 0.04–0.31 µM) in non-Tg cultures, 0.05 µM (95%CI: 0.02–0.12 µM) in Tg and 0.02 µM (95%CI: 0.01–0.04 µM) in Tg/Tg with a statistically significant difference among genotypes (F_2,284_ = 5.66, *P* = 0.0039).

Analysis of MPP^+^ effect on MAP-2-IR cell number revealed significant effect of MPP^+^ concentration (F_2,236_ = 5.89, *P*<0.0007), but neither a genotype effect (F_2,236_ = 0.27, *P* = 0.76) nor significant interaction between these two factors (F_2,236_ = 0.06, *P* = 1) (Fig. 9B).

These data indicate that dopaminergic tolerance to MPP^+^ was further reduced when basal urate content was reduced both in neurons and astrocytes.

## Discussion

In our model we induced selective degeneration of dopaminergic neurons using the neurotoxin MPP^+^ in mouse ventral mesencephalon cultures. Urate, a known powerful antioxidant [7]–[9], added to cultures 24 hours before and during toxicant treatment, attenuated MPP^+^ toxicity in dopaminergic neurons. It increased the number of TH-IR cells both in neuron-enriched and neuron-astrocyte cultures containing respectively low and high percentage of astrocytes. In cultures with low percentage of astrocytes, urate only partially prevented dopaminergic neuron loss. On the other hand, in cultures prepared with a high percentage of astrocytes, urate completely prevented MPP^+^-induced toxicity. Moreover, in these mixed neuron-astrocyte cultures, urate fully prevented atrophic morphological changes in neurite length and soma size induced by MPP^+^. Both in neuron-enriched and neuron-astrocyte cultures, urate showed protective effects with an EC_50_ of about 1 µM, a concentration within the mouse physiological range where its CSF urate concentration is about 3 µM [38], ten-time lower than in humans [6].

Urate may have conferred protection against neuronal atrophy and death through its established antioxidant actions, as it has been shown to prevent ROS accumulation and oxidative damage in other neuronal populations [25], [26], [39] and to raise cysteine uptake and glutathione synthesis in mouse hippocampal slices [40]. Urate treatment might change the redox status of neurons, reducing their vulnerability to oxidative stress and preventing cellular degeneration. In fact, MPP^+^ toxicity may depend on the antioxidant status of neurons. Previous studies have shown non-toxic levels of iron and glutathione synthesis inhibition to enhance degeneration of dopaminergic neurons treated with MPP^+^ [41] and antioxidant enzymes to prevent MPP^+^-induced toxicity [41]–[44].

Although its antioxidant properties have been extensively described, a question remains to be answered: How does *exogenous* urate prevents oxidant toxicity? Ascorbate, an important antioxidant in the CNS [45]–[47], is present at high levels in neurons where its concentration is thought to be raised and maintained by the sodium-dependent vitamin C transporter-2 (SVCT2) [48], [49]. Urate may protect through a similar mechanism that relies on the elevation of intracellular antioxidant content as was demonstrated here with *exogenous* urate substantially increasing intracellular urate in cortical neuronal cultures. By contrast, although Guerreiro et al. [25] reported a similar protective effect of urate on dopaminergic neurons in primary cultures, they did not find an associated increase in intracellular urate, possibly due to a greater sensitivity of our electrochemistry-based analytical methods or other differences between our studies.

To directly address the hypothesis that endogenous urate contributes to dopaminergic neuron resistance to toxicants, MPP^+^ toxicity was assessed in cultures expressing the UOx enzyme, which catalyzes urate degradation to allantoin. UOx is not normally synthesized in the mouse brain where, like in humans, urate is the enzymatic end product of the purine catabolism. Transgenic *UOx* expression reduced basal levels of urate both in cortical neurons and cortical astrocytes, even if this effect was not proportional to the increasing levels of UOx protein expression and enzyme activity observed with increasing transgene copy number. In neuron-enriched cultures, dopaminergic neurons expressing UOx were slightly more vulnerable to MPP^+^ compared to wild-type neurons. In neuron-astrocyte cultures, transgenic *UOx* markedly exacerbated the toxicity and increased the potency of MPP^+^ even though we did not see a greater decrease in intracellular urate concentration in astrocytes than in neurons. Because we were not able to measure urate content in ventral mesencephalic astrocytes and dopaminergic neurons due to their low number, we employed their cortical counterparts. Although urate transporter properties for each cell type are not expected to differ across brain regions, we cannot be sure that the changes in intracellular urate demonstrated in cortical cultures after both pharmacologic and genetic manipulations were achieved in ventral mesencephalic cells as well. Moreover, we cannot exclude that culturing neurons with astrocytes might affect the intracellular urate content in neurons. Nevertheless, our consistent observation of potentiated protection by astrocytes in cultures of dopaminergic neurons strengthens the evidence for a facilitative role of astrocytes on the neuroprotective effect of urate [35].

The small protective effect of urate in neuron-enriched cultures containing few astrocytes and the far greater protection in neuron-astrocyte cultures may reflect the same astrocyte-dependent mechanism in both culture types. This interpretation is supported by the absolute astrocyte dependence previously observed for urate's protective effect in spinal cord cultures [35]. Although the content of astrocytes and other dividing glial cell populations was pharmacologically reduced in our preparation of neuron-enriched cultures, astroglia was not completely eliminated from these cultures. Indeed a small astrocyte-independent effect of urate acting directly on dopaminergic neurons cannot be excluded with the available data.

How physiological levels of urate in astrocytes might play an important role in dopaminergic neuron protection is not known. It has been suggested that urate may confer neuroprotection via astrocytes by stimulating their extracellular glutamate buffering capacity or their release of neurotrophic factors [35], [50]. An intracellular antioxidant effect of urate on astrocytes might activate such glial functions. Indeed astrocytes were found to be susceptible to MPTP/MPP^+^ treatment showing increased ROS level [51], [52] and reduced glutamate buffering capacity [53], [54]. Therefore, even though the toxicant concentration we employed was selected to be subthreshold for altering astroglial viability, reducing basal levels of urate in astrocytes might deplete their antioxidant reserves and indirectly enhance toxic MPP^+^ effects on neurons. Although urate was found to protect neurons in association with the up-regulation of EAAT1 glutamate transporter expression in astrocytes [35], glutamate release was not detected in the striatum of MPP^+^-perfused mice [55] and NMDA antagonism did not prevent MPP^+^-induced dopaminergic cell death [56]. Further experiments will be needed to clarify the mechanism by which astrocytes play a facilitative role in the neuroprotective effect of urate and to confirm urate's protective effect in animal models of PD.

In conclusion, our data showed that intracellular urate may modulate dopaminergic neuron resistance to environmental toxins. This effect may be mediated by changes in astroglial urate content. A greater understanding of how urate protects neurons in models of PD may not only help elucidate its pathophysiology, it may also help accelerate or refine current urate-targeting strategies under investigation for their potential to slow or prevent PD (http://clinicaltrials.gov/ct2/show/NCT00833690).

## Materials and Methods

### Mice

UOx Tg mice [37] were obtained from Kenneth L. Rock at University of Massachusetts. Mice were backcrossed eight times on the C57BL/6 genetic background and phenotyped by measuring UOx activity in serum samples. Briefly, about two hundred µl of submandibular blood were collected from 1 month-old mice. Four µl of serum sample were added to 96 µl of 130 µM urate in 0.1 M borate (pH 8.5) and absorbance was read at 292 nm at the beginning of the assay and after 4–6 hours incubation at 37°C.

### Ethics Statement

All experiments were performed in accordance with the National Institutes of Health Guide for the Care and Use of Laboratory Animals, with approval from the animal subjects review board of Massachusetts General Hospital (Permit Number: 2006N000120).

### Neuron-enriched cultures

Ventral mesencephalon was dissected from embryonic day E15–17 mouse embryos. Tissue was carefully stripped of their meninges and digested with 0.6% trypsin for 15 min at 37°C. Trypsinization was stopped by adding an equal volume of culture preparation medium (DMEM/12, N2 supplement 5%, fetal bovine serum (FBS) 10%, penicillin 100 U/ml and streptomycin 100 µg/ml) to which 0.02% deoxyribonuclease I was added. The solution was homogenized by pipetting up and down, pelleted and re-suspended in culture medium (Neurobasal medium (NBM), B27 supplement 2%, L-gluatamine (2 mM), penicillin 100 U/ml and streptomycin 100 µg/ml). The solution was brought to a single cell suspension by passage through a 40-µm pore mesh. Cells were seeded at a density of 220,000 cells/cm^2^ onto 96 well plates or chamber-slides coated with poly-L-lysine (100 µg/ml)/DMEM/F12) and cultured at 37°C in humidified 5% CO2-95% air. On the third day half medium was replaced with fresh NBM containing the antimetabolite cytosine arabinoside (Ara-C,10 µM) to inhibit glial growth and glucose 6 µM. Medium was fully changed after 24 hours and then a half volume was replaced every other day. After 6 days in vitro (DIV) cultures were pretreated with urate or vehicle, and 24 hours later MPP^+^ or vehicle was added. Cultures were constitute of >95% neurons, of which 2–3% were dopaminergic neurons and <5% astrocytes; microglia and oligodendrocytes were not detected (See Figure 1).

For Tg neuronal cultures, individual cultures were prepared from the ventral mesencephalon of individual embryos generated by crossing two Tg mice (with a resulting distribution of 23% non-Tg, 44% Tg and 33% Tg/Tg). The rest of the brain was used for phenotyping by western blotting. Brain tissue extracts negative for UOx staining were considered non-Tg; tissue, positive for UOx staining were considered Tg when UOx/actin value was >0.1 and ≤0.6, and Tg/Tg when UOx/actin value was >1.5. Cultured cell phenotypes were confirmed by measuring UOx activity in the cell medium.

### Neuron-astrocyte cultures

Tissue was processed as described above but no Ara-C was added to the cultures. At 4 DIV cells were treated with urate or vehicle, 24 hour later MPP^+^ or vehicle was added. Cultures comprised 45–60% neurons, of which 2–3% were dopaminergic neurons, 40–50% astrocytes and <1 microglia, oligodendrocytes were not detected.

**Immunocytochemistry:** After treatments cultures were fixed with 4% paraformaldehyde for 1 hour at room temperature. Then, cells were loaded with a blocking solution (0.5% albumin, 0.3% Triton-X 100 in phosphate buffer saline) for 30 min at room temperature and then incubated with a mouse anti-TH (1∶200, Millipore, Temecule, CA) and a rabbit anti-MAP-2 antibody (1∶200, Millipore, Temecule, CA), or a rabbit anti-GFAP antibody, overnight at 4°C to label dopaminergic neurons neurons and astrocytes, respectively. Cultures were loaded with a cy3-conjugated anti-mouse antibody (1∶500, Jackson InnunoResearch Laboratories, Inc.; West Grove, PA) and a FITC-conjugated anti-rabbit antibody (1∶300, Jackson InnunoResearch Laboratories, Inc.; West Grove, PA) 2 hours at room temperature. Cultures were imaged using an Olympus BX50 microscope with a 20×/0.50 objective and Olympus DP70 camera. Images were processed with DP Controller software (Olympus) and merged with ImageJ (NIH). Cells cultured in plates were observed with a Bio-Rad Radiance 2100 confocal laser-scanning microscope with krypton-argon and blue diode lasers. Images were acquired through a Plan Fluor DIC ELWD 20×/0.45 Ph1 DM ∞/0–2 WD 7.4 objective on an inverted Nikon Eclipse TE300 fluorescent microscope with 408/454 nm excitation/emission (blue), 485/525 nm excitation-emission (green) and 590/617 nm excitation-emission (red).

Neurite length was measured by the Simple Neurite Tracer tool of ImageJ software. In each sample, neurite length was determined by the average of the longest neurite of 100 TH-IR neurons randomly selected in the well. Neurons having neurites ending outside the optic field were excluded from the analysis. Values were expressed in µm.

### High-Performance Liquid Chromatography

Cells were scraped in a solution of 150 mM phosphoric acid, 0.2 mM EDTA, and 1 µM 3,4-dihydroxybenzylamine (DHBA; used as internal standard), clarified by centrifugation and filtered through a 0.2 µm Nylon microcentrifuge filter (Spin-X, Corning). Samples were chromatographed by a multi-channel electrochemical/UV HPLC system with effluent from the above column passing through a UV-VIS detector (ESA model 528) set at 254 nm and then over a series of electrodes set at −100 mV, +250 mV and +450 mV. Urate was measured on the +250 mV electrode with a limit of detection at 0.0001 mg/dl. In order to generate a gradient, two mobile phases were used. Mobile phase B increased linearly from 0% to 70% between 6^th^ and 14^th^ min of the run and immediately reduced to 0% at 17.4 min and allowed to re-equilibrate for the final 3.6 min. Mobile phase A consisted of 0.2 M potassium phosphate and 0.5 mM sodium 1-pentanesulfonate; mobile phase B consisted of the same plus 10% (vol/vol) acetonitrile. Both mobile phases were brought to pH 3.5 with 85% (wt/vol) phosphoric acid.

### Western blot assay

Cells were scraped in RIPA buffer (Sigma Co., St. Luis, MO) and loaded (50 µg of proteins per well) into a 10% SDS-PAGE gel. Proteins were then transferred electrophoretically onto 0.2 µ nitrocellulose membranes (Biorad Laboratories) and probed with a rabbit polyclonal antibody anti-UOx (1∶200; Santa Cruz, CA) overnight at 4°C. After washing in Tris Buffer Saline containing 0.1% Tween20, membranes were incubated with a horseradish peroxidase-conjugated anti-rabbit IgG (1∶2000; Pierce, Biotechnology, Rockford, IL, USA) for 2 hours at room temperature. Proteins were visualized using chemiluminescence (Immobilon, Millipore). In order to normalize the values of UOx staining, β-actin was detected in the same western blot run. Membranes were incubated for 2 hours at room temperature with an anti-β-actin antibody (1∶2000; Sigma, St Louis, MO) and then with a horseradish peroxidase-conjugated anti-rabbit IgG (1∶5000; Pierce, Biotechnology, Rockford, IL) for 2 hours. Membranes were developed as above. Bands were acquired as JPG files and densitometric analysis of bands was performed by ImageJ software. UOx/β-actin values were expressed as arbitrary units.

### UOx activity assay

Cell medium was added to 0.5 mg/ml urate and absorbance was red at 292 nm before and after 24 hours incubation at 37°C. Activity was calculated as percentage of absorbance decrease in comparison to starting values.

**Protein detection:** Proteins were quantified in 4 µl of each sample using Bio-Rad Protein Assay reagent (Bio-Rad, Hercules, CA, USA) and measured spectrophotometrically at 600 nm with Labsystems iEMS Analyzer microplate reader.
